# A novel dipeptide from potato protein hydrolysate augments the effects of exercise training against high-fat diet-induced damages in senescence-accelerated mouse-prone 8 by boosting pAMPK / SIRT1/ PGC-1α/ pFOXO3 pathway

**DOI:** 10.18632/aging.103081

**Published:** 2020-04-26

**Authors:** Shibu Marthandam Asokan, Ting Wang, Ming-Fu Wang, Wan-Teng Lin

**Affiliations:** 1 Cardiovascular and Mitochondria Related Disease Research Center, Buddhist Tzu Chi Hospital, Hualien, Taiwan; 2 Graduate Institute of Biomedical Sciences, China Medical University, Taichung, Taiwan; 3 Department of Hospitality Management, College of Agriculture, Tunghai University, Taichung, Taiwan; 4 Department of Food and Nutrition, Providence University, Taichung, Taiwan; 5 Department of Senior Wellness and Sport Science, College of Agriculture, Tunghai University, Taichung, Taiwan

**Keywords:** bioactive peptides, alcalase, longevity, cardio-protection, hepato-protection

## Abstract

The pathological effects of obesity are often severe in aging condition. Although exercise training is found to be advantageous, the intensity of exercise performed is limited in aging condition. Therefore in this study we assessed the effect of a combined treatment regimen with a short-peptide IF isolated from alcalase potato-protein hydrolysates and a moderate exercise training for 15 weeks in a 6 month old HFD induced obese senescence accelerated mouse-prone 8 (SAMP8) mice model. Animals were divided into 6 groups (n=6) (C:Control+BSA); (HF:HFD+BSA); (EX:Control+ BSA+Exercise); (HF+IF:HFD+ IF); (HF+EX:HFD+Exercise); (HF+EX+IF:HFD+Exercise+IF). A moderate incremental swimming exercise training was provided for 6 weeks and after 3 weeks of exercise, IF was orally administered (1 mg/kg body Weight). The results show that combined administration of IF and exercise provides a better protection to aging animals by reducing body weight and regulated tissue damage. IF intake and exercise training provided protection against cardiac hypertrophy and maintains the tissue homeostasis in the heart and liver sections. Interestingly, IF and exercise training showed an effective upregulation in pAMPK/ SIRT1/ PGC-1α/ pFOXO3 mechanism of cellular longevity. Therefore, exercise training with IF intake is a possible strategy for anti-obesity benefits and superior cardiac and hepatic protection in aging condition.

## INTRODUCTION

Aging is a natural process denoted by a progressive decline in normal physiological ability and increase in age-associated disorders [1, 2]. Aging is frequently correlated as one of the key risk factor for metabolic syndromes and cardiovascular disorders. Incidence of obesity and insulin resistance in aging population is common and is a contributing factor of major health issues among the elderly [3, 4]. Obesity, in particular, inflicts heavy burden on the normal life and function of an individual, with more than 650 million of obese adults it has emerged as a major medical challenges of the century [5–7]. Liver being the principle organ in systemic maintenance of metabolic homeostasis and its dysfunction, as in the case of nonalcoholic fatty liver disease (NAFLD), plays a major role in the progression of metabolic disorders. Hepatosteatosis, a reversible initial stage of NAFLD, may further develop into nonalcoholic steatohepatitis (NASH), fibrosis and liver cirrhosis [8, 9]. In the steatosis stage of NAFLD the hepatic accumulation of triglyceride increases lipotoxicity in the liver [10, 11]. Circulating free fatty acids (FFAs) are the important contributor of liver triacylglycerol (TG) content in the non-adipose tissues and the plasma FFA contents are often associated with severity of NAFLD [12, 13]. Several studies have revealed cardiovascular disease as one of the most prevalent causes of morbidity and mortality among NAFLD patients [14–18]. Excessive FFA import into cardiomyocytes and subsequent accumulation causes lipotoxicity in cardiomyocytes and results in apoptosis of heart cells.

Obese conditions are also known to trigger structural and functional variations in heart in humans as well as in animal models. Obesity is associated with cardiac hypertension, increased incidences of chronic volume overload and left ventricular hypertrophy [19–21]. The cardiac effects of obesity are aggravated with aging due to systemic dysregulation in metabolism. Further, previous reports also reveal that aging aggravates the incidence of NAFLD and the prevalence of NAFLD among those aged above 65 years of age is around 35%. Aging also increases the susceptibility for high fat diet feeding associated hepatic fibrosis, which is also correlated with other organ damages [22].

Regular physical exercise is widely known to improve human physiological function, prevent metabolic disorders and to reduce the risks of ageing associated diseases such as cardiovascular diseases, type 2 diabetes, hypertension, dyslipidemia, and cancer [23, 24]. In our previous studies, exercise training was found to increase the AMPK/SIRT1/PGC-1α/FOXO3 signaling mechanism in the gastrocnemius muscles, soleus muscles and cardiac muscle. In addition, exercise training is also found to provid protection against apoptosis of heart and muscle cells in aging mice [23, 25–27]. But the effect of exercise training on NAFLD associated hepatic and cardiac abnormalities are not very clear yet. Interestingly, our recent studies reveal that alcalase derived potato protein hydrolysates (APPH) with lipolysis-stimulating activity and their derivatives were found to possess efficient anti-obesity and cyto-protective potentials [1, 26, 28–31]. To investigate the modalities of fatty liver diseases and potential therapeutic interventions various diet based murine models were used in previous studies that elucidated the therapeutic potentials of protein hydrolysates from soy or potato [1, 32, 33]. Contemplating the dynamics of drug administration in aging people, we carried out the present study by inducing NAFLD in senescence accelerated mouse-prone 8 (SAMP8) mice strains with HFD feeding [34, 35]. Supplementing HFD to SAMP8 mice models can properly mimic the aging associated effects in NAFLD pathogenesis.

**Figure 1:**
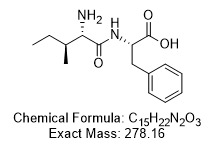
**Chemical structure of IF peptide.** Amino acid arrangement of the bioactive peptide IF isolated from APPH potato-extract hydrolysate generated by alcalase treatment.

With precedents from our previous studies, we presume that an integrative approach with exercise training and potato-protein hydrolysates administration may exhibit a synergistic protective effect against obesity-associated effects in heart and liver. While APPH is a lipolysis stimulating peptide with efficient anti-obesity effects, IF a short peptide (Figure 1) containing only two amino acids isoleucine (I) and phenylalanine (F) has been observed very frequently in APPH preparation [30]. The in vitro cytoprotective benefit of IF has been previously determined under hyperglycemic condition. Therefore, in this study we further study we elucidate the anti-obesity effects of IF in HFD fed aging models and further determined the synergistic effects of IF with respect to exercise training.

## RESULTS

### HFD inflicted changes in body weight, epididymal fat weight, liver weight and cardiac characteristics

**Figure 2:**
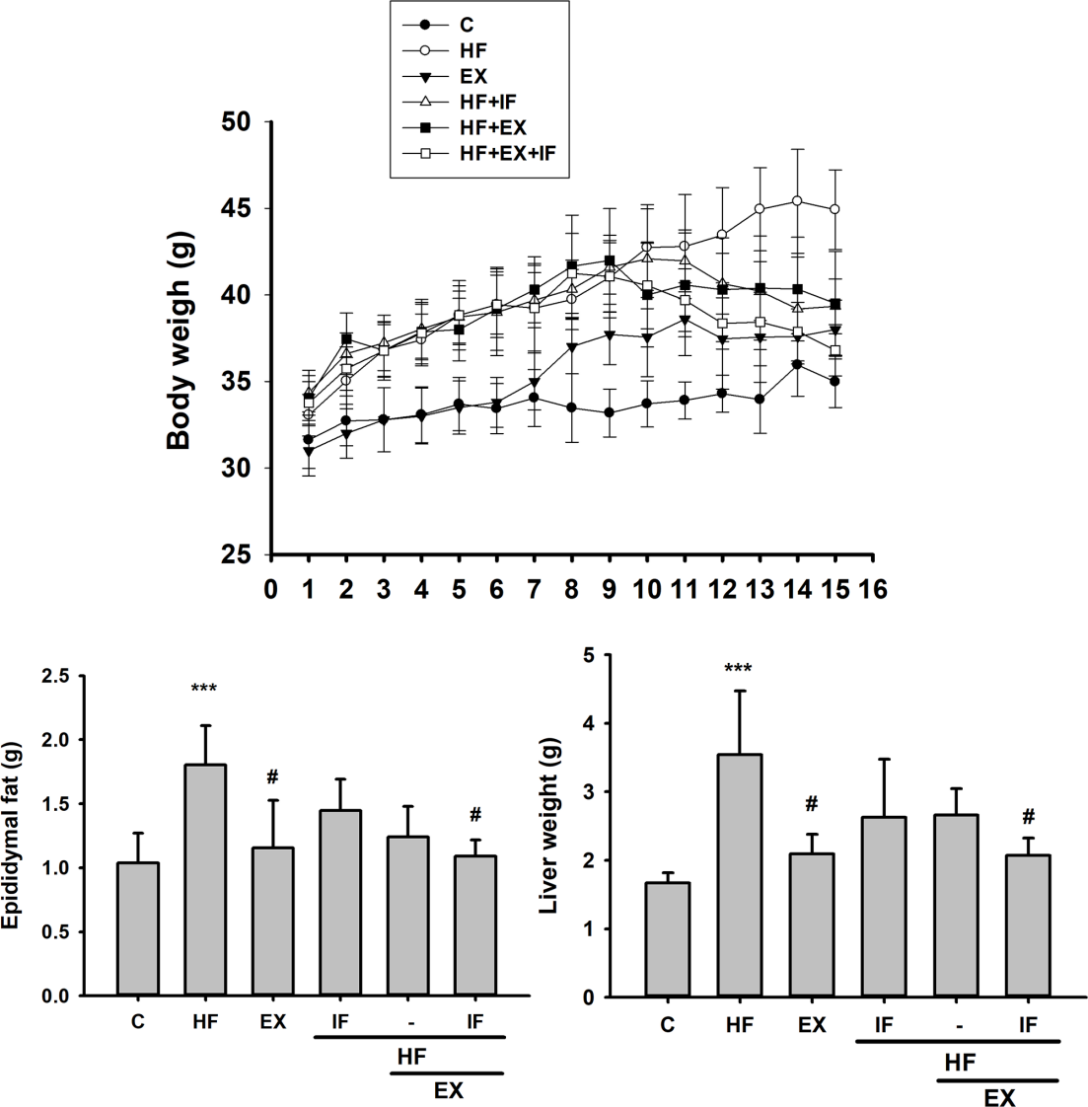
**IF treatment with exercise training show better anti-obesity effects.** Measurement on the Body Weight, Epididymal Fat Mass, and Liver Weight after IF administration and 15 weeks of exercise training show variation among SAMP8 mice from different group (n=6); C: Control; HF: High-fat diet; EX: Exercise; HF+IF: High-fat diet+IF; HF+EX: High-fat diet+ Exercise; HF+EX+IF: High-fat diet+ Exercise+ IF. Bars indicate the mean ± SEM obtained from experiments performed in triplicate. ^***^P<0.001 compared with the control group, *^#^P*<0.05 compared with the HF group.

As shown in Figure 2, HFD intake triggered several characteristic obesity associated changes in the test mice. The mice fed with HFD showed considerable increase in body, liver and epididymal fat weight when compared to the control group. However, after 8-weeks of exercise, the HF+EX group showed a notable decrease in the body weight, epididymal fat weight and liver weight when compared to HF group. Interestingly, IF+HF+EX showed a better reduction body weight, epididymal fat weight and liver weight signifying anti-obesity effects.

Moreover, the heart weight also showed notable differences between each group. The whole heart weight (WHW), WHW/tibia increased in the HF group when compared to the control group (*p*<0.05). IF peptide treatment, alone or in combination with exercise for 8 weeks significantly decreased the HFD effects compared to those in HF group (*p*<0.05) (Table 1). These results indicated that the IF treatment was effective in the HFD-induced aging rat model.

**Table 1:** Cardiac characteristics of the 6 groups (n = 6).

	**Control**	**High-fat diet**	**Exercise**	**High-fat diet + IF**	**High-fat diet + Exercise**	**High-fat diet + Exercise + IF**
**Whole heart weight (WHW), g**	0.245±0.045	0.318±0.009*	0.275±0.032^#^	0.263±0.178^#^	0.302±0.025	0.240±0.028^#^
**Tibia (mm)**	2.100±0.071	2.080±0.042	2.213±0.054	1.993±0.033	1.970±0.000	2.040±0.057
**WHW/tibia, g/mm**	0.116±0.018	0.153±0.001*	0.124±0.015^#^	0.132±0.008	0.150±0.008	0.118±0.017^#^

Abbreviations: WHW, whole heart weight; WHW/tibia, whole heart weight normalized by tibia length.

Values are mean ±S.D. *P<0.05, compared with control group; *^#^P*<0.05, compared with High-fat diet group. *indicates a significant difference from the control group at *P*<0.05. ^#^indicates a significant difference from the High-fat diet group at *P*<0.05.

### Effect of IF and exercise on serum biochemical markers associated with obesity

**Figure 3:**
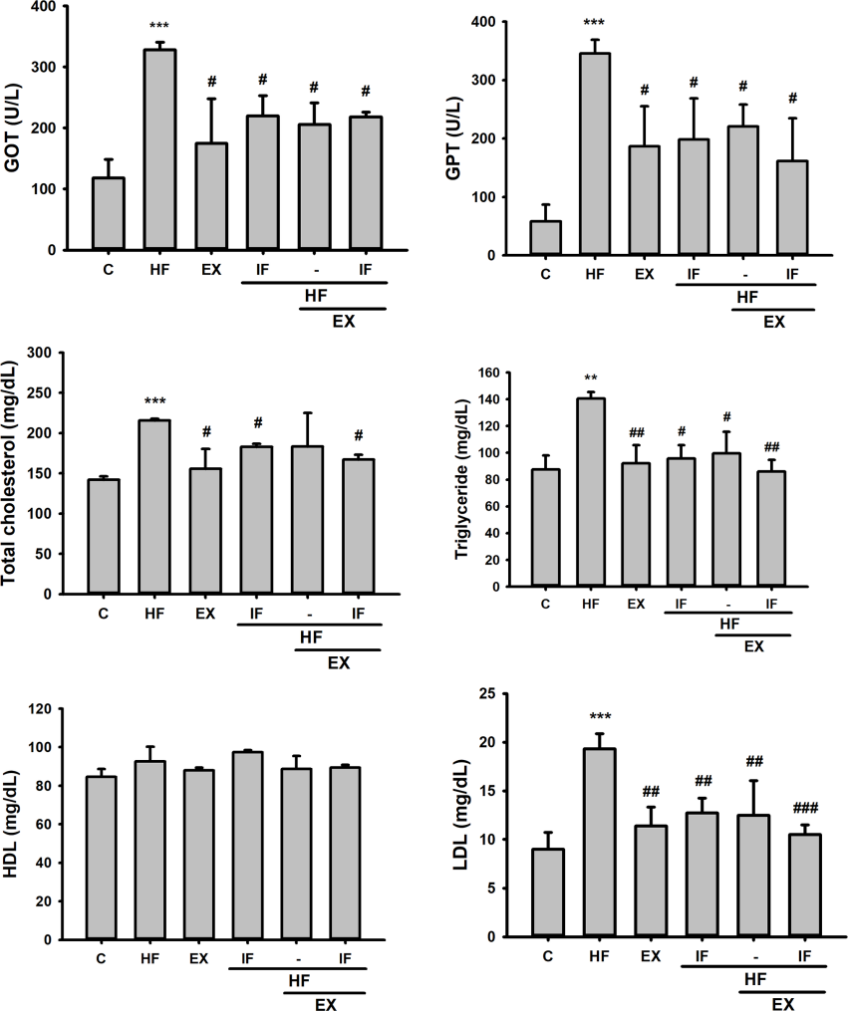
**Effect of IF and Exercise on Serum biochemical markers for heart and liver tissue damages.** Biochemical analysis after IF administration and 15 weeks of exercise training show the difference in plasma glutamic oxaloacetic transaminase (GOT) and glutamic pyruvic transaminase (GPT) levels and serum levels of total cholesterol, Triglycerides, high-density lipoprotein cholesterol (HDL-C), low-density lipoprotein cholesterol (LDL-C) and blood glucose among the different SAMP8 mice groups (n=6). C: Control; HF: High-fat diet; EX: Exercise; HF+IF: High-fat diet+IF; HF+EX: High-fat diet+ Exercise; HF+EX+IF: High-fat diet+ Exercise+ IF. Bars indicate the mean ± SEM obtained from experiments performed in triplicate. ^**^P<0.01, ^***^P<0.001 compared with the control group, *^#^P*<0.05, *^##^P*<0.01 compared with the HF group.

The serum lipid profile analysis showed high serum levels of liver and heart tissue damage markers such as GOP, GTP, and obesity associated LDL, TC and TG in the aging mice on HFD feeding. However, GOT, GPT, TG, TC, and LDL levels were effectively reduced by IF treatment alone or along with exercise training in the HFD fed SAMP8 mice models (Figure 3). The results show that IF administration and swimming exercise training equally regulate serum cholesterol levels and provide cardio-/hepato-protection against obesity effects in aging animals.

### Ameliorating effects of IF and exercise on HFD induced changes in heart and liver tissue morphology

**Figure 4:**
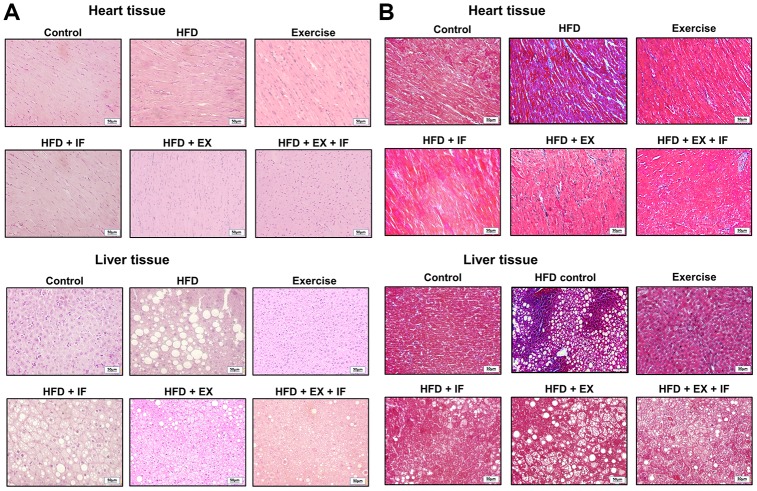
**IF peptide administration in combination with exercise training on HFD induced heart and liver histology in aging model.** Hematoxylin and eosin (**A**) and Masson’s trichrome (**B**) staining on heart and liver tissues obtained after IF administration and 15 weeks of exercise training show difference in liver and heart histology among different groups (n=6) of SAMP8. C: Control; HF: High-fat diet; EX: Exercise; HF+IF: High-fat diet+IF; HF+EX: High-fat diet+ Exercise; HF+EX+IF: High-fat diet+ Exercise+ IF. Scale bars, 50 μm. (Magnification, 200x).

The histological analysis of cardiac and hepatic tissue sections show that HFD feeding in SAMP8 mice inflicts obvious changes in the tissue characteristics and architecture (Figure 4). As seen from the Hematoxylin and Eosin (H & E) stained tissue sections (Figure 4A), myocardial disarray in HFD group hearts characterized by increase in interstitial spaces and peculiar arrangement of cardiomyocytes were confirmed (Figure 4A). The liver tissue section of HFD group mice show loosely arranged hepatic cords; dilated sinusoid, hepatocytes vacuolation, and multi-focal necrosis. In addition Masson's trichrome stain on the heart and the liver tissue show collagen accumulation in the interstitial spaces (Figure 4B). However, these pathological changes were effectively reversed by 8-weeks exercise training (Figure 4). Moreover, the results also convey that IF administration further enhances the beneficial effects of exercise in the aging heart and liver.

### IF administration improved exercise triggered alleviation of HFD induced apoptosis

**Figure 5:**
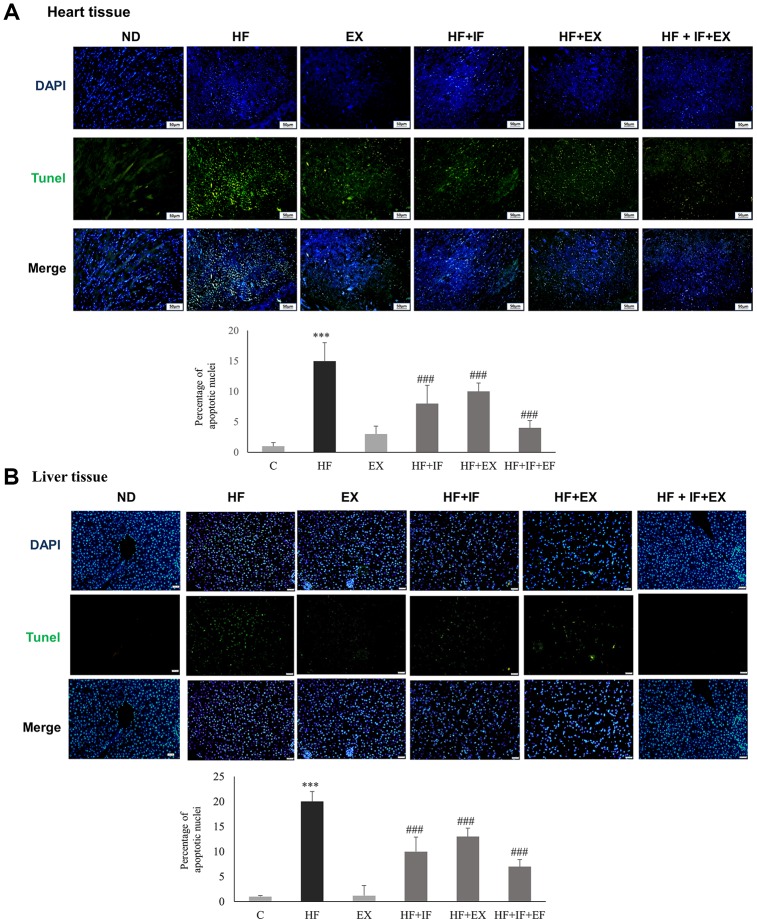
**IF administration during exercise show better protection from HFD induced hepatic and cardiac apoptosis in aging accelerated SAMP8 mice.** TUNEL staining show the difference in TUNEL positive cells induced by HFD feeding in heart (**A**) and liver (**B**) tissues in different groups (n=6) of SAMP8 mice. The nucleus are stained in blue and the TUNEL positive apoptotic nuclei are stained in green. C: Control; HF: High-fat diet; EX: Exercise; HF+IF: High-fat diet+IF; HF+EX: High-fat diet+ Exercise; HF+EX+IF: High-fat diet+ Exercise+ IF. Scale bars, 200 μm. (Magnification, 200x). Bars indicate the mean ± SEM obtained from experiments performed in triplicate. ^***^P<0.001 compared with the control group, *^###^P*<0.001 compared with the HF group.

**Figure 6:**
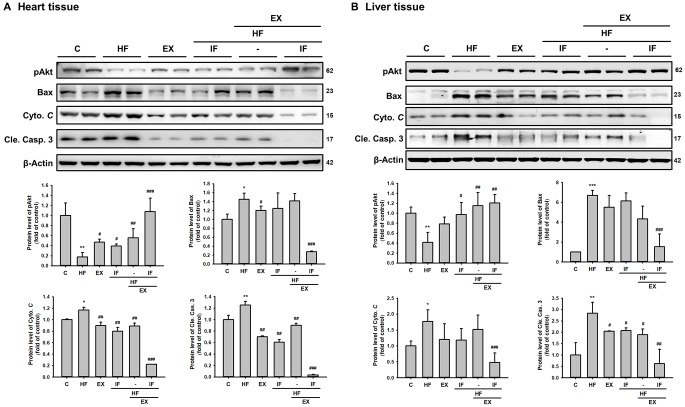
**Combined administration of IF peptide and exercise show better regulation in the molecular events of cell survival and apoptosis.** The levels of survival and apoptosis protein such as pAkt, Bax, Cyto *C* and caspase 3 in different groups (n=6) of aging SAMP 8 mice (**A**) heart and (**B**) liver. All protein samples from each rat group were analyzed by Western blotting. The protein expression folds were normalized with β-actin. C: Control; HF: High-fat diet; EX: Exercise; HF+IF: High-fat diet+IF; HF+EX: High-fat diet+ Exercise; HF+EX+IF: High-fat diet+ Exercise+ IF. Bars indicate the mean ± SEM obtained from experiments performed in triplicate. ^*^P<0.05, ^**^P<0.01, ^***^P<0.001 compared with the control group, *^#^P*<0.05, *^##^P*<0.01, *^###^P*<0.001 compared with the HF group.

We further investigated the effect of IF administration and exercise training on cellular apoptosis in cardiac tissues. TUNEL staining of the respective tissue sections showed that HFD feeding caused significant apoptosis in the heart tissues, compared with the control group (Figure 5). The results showed the protective effect of IF treatment and exercise training on HFD induced apoptosis. The significant increase in the TUNEL positive nuclei observed in the HF group was found to be attenuate in the IF treatment group and in the exercise trained group mice. Interestingly the mice that received both IF and exercise training exhibited a better protection against HFD induced apoptosis. Therefore, IF feeding during exercise training regimen would help in better cardio-protection in SAMP8 mouse models. Further confirmation of the results using Western blotting showed that the anti-apoptotic protein pAkt was significantly upregulated and the apoptotic proteins such as Bax, cytochrome *c* and cleaved caspase 3 were significantly increased in the HF group mice however, IF administration and exercise treatment effectively reversed the HFD induced effects in both heart and liver tissues of SAMP8 mice (Figure 6). The results also confirmed that combined treatment of IF and exercise show better protection in heart and liver tissues.

**Figure 7:**
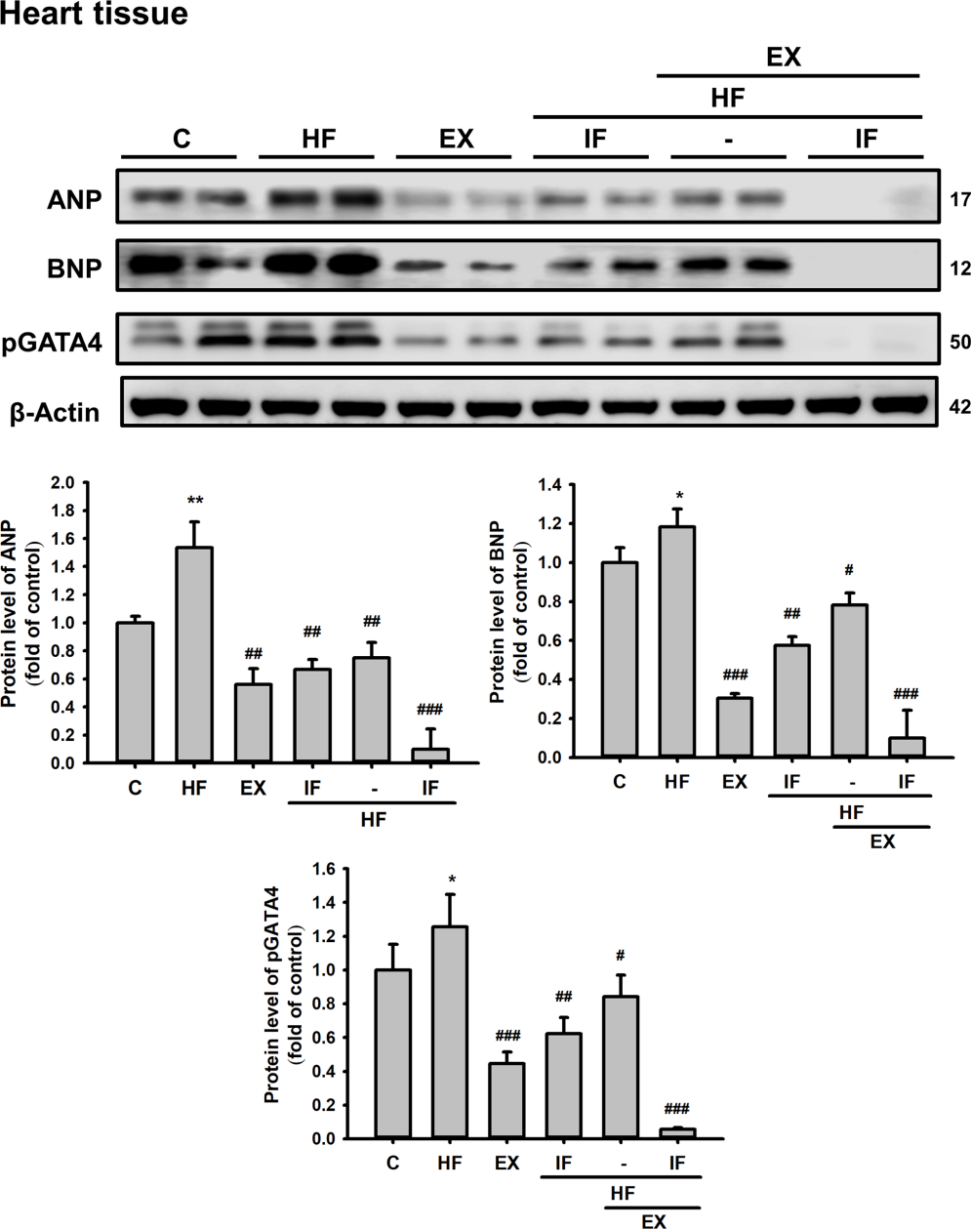
**Combined administration of IF peptide and exercise show better regulation of HFD induced cardiac hypertrophic markers in SAMP8 aging model.** The levels of survival and apoptosis protein such as ANP, BNP, and pGATA4 in aging mice heart show. All protein samples from each rat group were analyzed by Western blotting (n = 6). The protein expression folds were normalized with β-actin. C: Control; HF: High-fat diet; EX: Exercise; HF+IF: High-fat diet+IF; HF+EX: High-fat diet+ Exercise; HF+EX+IF: High-fat diet+ Exercise+ IF. Bars indicate the mean ± SEM obtained from experiments performed in triplicate. ^*^P<0.05, ^**^P<0.01 compared with the control group, *^#^P*<0.05, *^##^P*<0.01, *^###^P*<0.001 compared with the HF group.

Further analysis in cardiac tissue also showed that IF and exercise treatment suppresses HFD induced expression of hypertrophic marker such as ANP and BNP and also hypertrophy associated transcription factor p-GATA4 (Figure 7).

### IF augments pAMPK/SIRT1/PGC1α associated longevity mechanism in exercise trained SAMP8 mice heart and liver tissue.

**Figure 8:**
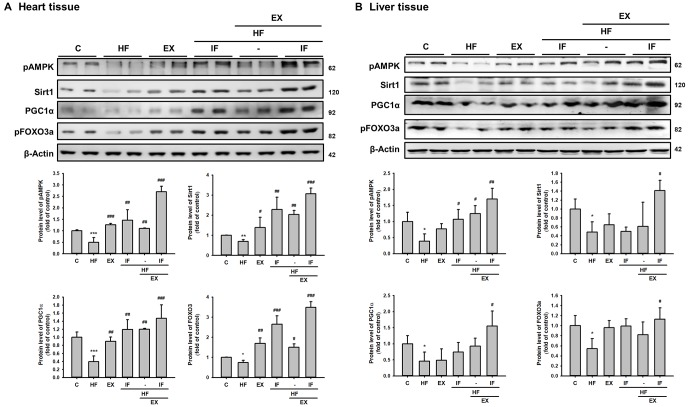
**Combined treatment with IF peptide and swimming exercise provide better cardiac and liver rejuvenation potential against HFD effects by enhancing pAMPK/ SIRT1/ PGC-1α/ pFOXO3 protein expression in SAMP8 aging model.** The levels of survival and apoptosis protein such as pAMPK/ SIRT1/ PGC-1α/ pFOXO3 in aging rat (**A**) heart and (**B**) liver tissue. C: Control; HF: High-fat diet; EX: Exercise; HF+IF: High-fat diet+IF; HF+EX: High-fat diet+ Exercise; HF+EX+IF: High-fat diet+ Exercise+ IF. Bars indicate the mean ± SEM obtained from experiments performed in triplicate. ^*^P<0.05, ^**^P<0.01 compared with the control group, *^#^P*<0.05, *^##^P*<0.01, *^###^P*<0.001 compared with the HF group.

To investigate the role of IF and exercise on longevity mechanism in HFD fed SAMP8 mice the protein levels of pAMPK, SIRT1, PGC-1α were analyzed by Western blotting. As seen in the representative Western blot of pAMPK, SIRT1, PGC-1α and pFOXO3a levels in both heart and liver showed significantly reduction. However, the mice in the EX+HF groups and IF+HF showed a significantly increased protein levels of pAMPK, SIRT1, PGC-1α and pFOXO3a when compared to the HF groups. Interestingly, the protein level in IF+HF+EX group was more significantly increased than HF group (Figure 8). Therefore, IF treatment potentially augments the effects of exercise in promoting SIRT1 associated longevity signaling in HFD fed aging mice and thereby provides enhanced cardiac and hepatic protection.

## DISCUSSION

Aging is accompanied by progressive deterioration of cellular biochemical functions such as oxidative phosphorylation and mitochondrial respiratory chain [36–38]. Decline in the mitochondrial function leads to accumulation of oxidative stress which results in senescence and modulations in the levels of longevity proteins like the SIRT1 [39, 40]. The redox-sensitive enzyme SIRT1, regulates an array of cellular functions such as cellular apoptosis and survival, endocrine signaling, chromatin remodeling, and gene expression [41]. Activation of SIRT1 results in deacetylation and transcriptional activation of PGC-1α which subsequently promotes mitochondrial biogenesis. It is known that increase in PGC-1α expression facilitates mitochondrial biogenesis and enhances oxidative phosphorylation in muscle, heart and fat tissues [42–45]. Also PGC-1α expression is essential for the antioxidant effects of exercise training that provides protection against aging-associated defects in mitochondrial proteins and cellular apoptosis [46–48]. SIRT1 therefore is an important mediator that replenishes cellular metabolism and suppresses inflammatory signaling [49, 50]. SIRT1 in heart is known to provide cardio-protection in conditions like hypertrophy and myocardial infarction [51–53]. SIRT1 is also involved in controlling hepatic lipid metabolism, moderating oxidative stress in liver and protection from HFD induced hepatic inflammation, glucose intolerance and hepatic steatosis [54, 55]. Therefore, therapeutic strategies to enhance SIRT1 levels during stress conditions could potentially provide protection to heart and liver tissue organization and function.

Senescence-accelerated mouse (SAM) model is an appropriate murine model to study aging that has a short lifespan and display senescence earlier. SAMP8 is a SAM strain considered in studying aging associated liver dysfunction [56, 57]. SAMP8 mice models reflect events associated with aging associated liver damages such as increased oxidative damage and increase in aspartate aminotransferase and alanine aminotransferase indicating damages to liver or muscle tissues. In addition it is well known that accelerated senescence in SAMP8 models trigger cardiac damages such as cardia fibrosis and diastolic dysfunction [58]. In addition it is well known that accelerated senescence in SAMP8 models trigger cardiac damages such as cardiac fibrosis and diastolic dysfunction [59]. Aging is well known to increase the susceptibility to HFD induced metabolic syndrome therefore in this study we used SAMP8 animals models that were fed with HFD as an animal model representing aggravated effects of HFD under aging condition [60].

Accumulating evidences suggest that exercise training augments cardio-protection and restores cardiac function against various challenges [61–63]. Exercise training is also known to attenuate hepatic lipid accumulation and provide anti-apoptosis and anti-fibrosis effects in HFD-fed mice signifying their therapeutic potential in the amelioration of NAFLD [23, 64–66]. Since cardiac events and disorders such as NAFLD are common in elderly and such pathological events also get aggravated with aging it is essential to find better therapeutic strategies to attenuate such complex conditions. While exercise training is known to ameliorate aging and obesity associated health issues, the potential to perform the required intensity and duration of exercise for therapeutic benefit varies between individual [67]. Further the reduced exercise tolerance associated with aging leads to structural and functional changes in the heart causing deterioration in cardiac function [68]. Therefore an integrated approach with low to moderate exercise training and a pharmacological intervention could be an efficient therapeutic strategy. Our previous studies demonstrate that resveratrol as a therapeutic intervention enhances the cardio-protective effects of exercise training by improving SIRT1 associated longevity mechanism [69]. Our previous studies have also demonstrated that APPH and its short peptide constituents such as IF and DIKTNKPVIF provide cytoprotection against aging and HFD induced damages by improving the longevity signal mechanism [57, 70, 30]. Therefore, in this study, an integrated approach with moderate, incremental exercise training and peptide administration was employed against HFD induced cardiac and hepatic damages in aging condition using SAMP8 mice model. The novelty of the present study resides in the first ever attempt to mimic NAFLD in SAMP8 strains for observing combined cardiac and hepatic effects. Although, various reports have suggested senescence-accelerated mice prone 8 as an appropriate aging model in NAFLD studies, there are no longevity associated studies performed yet to examine NAFLD using HFD-fed SAMP8 mice [57].

**Figure 9:**
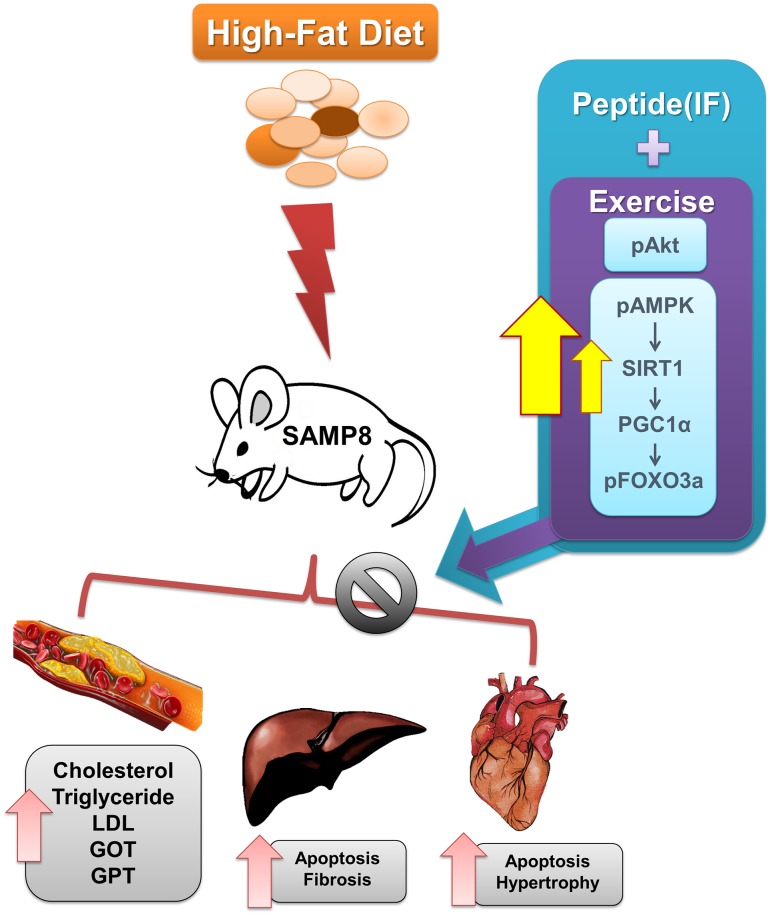
**Graphical representation of the mechanism by which IF and exercise fortify cardiac and hepatic protection against HFD induced damages in aging condition.**

The results show that administration of IF along with exercise training provide protection against HFD induced cardiac and hepatic cell death in SAMP-8 mice models with increase in SIRT1 levels (Figure 9). SIRT1 is directly implicated on the levels of SIRT3 whose functions appear reduced in aging and in obesity [27, 71].

The enhancement of SIRT1 following combined effects of exercise and IF treatment potentially lead to normal deacetylation and stability of SIRT3 protein and its function. SIRT1 increase has been shown to ameliorate NAFLD effects on liver, reduce body weight and suppress HFD induced inflammatory mediators. The peptide IF being a potent SIRT1 activator also blocks FOXO3 to inhibit FAS/FADD/Caspase8 involved extrinsic apoptosis. Similarly exercise training also attenuates FOXO3a levels with elevation in SIRT1 levels to provide cardio-protection. Therefore combined IF and exercise training is a promising strategy to treat functional limitations in late life.

In conclusion, the findings are substantial as complementing IF with exercise could potentially reduce the intensity of required exercise for protection from HFD associated aging effects. Our results provided clear in vivo experimental support for peptide with exercise has protected the cardiac and liver abnormality.

## MATERIALS AND METHODS

### Chemicals

All the chemicals used in the present study were analytical grade. The peptides were commercially synthesized by DG peptides CO., Ltd, China.

### Animal experiments

The animal use experimental protocol was approved by the Institutional Animal Care and Use Committee (IACUC) of Providence University. The study followed the principles of laboratory animal care (NIH publication). All of the SAMP8 Mice used were 6 months of age and were purchased from BioLASCO Taiwan Co., Ltd. (Taipei, Taiwan). After acclimatization for a week the animals were divided into 6 groups (C: Control + BSA); (HF: High-fat diet + BSA); (EX: Control + BSA + Exercise); (HF + IF: High-fat diet+ IF); (HF + EX: High-fat diet + Exercise); (HF + EX + IF: High-fat diet + Exercise + IF) (n = 6 each) first groups were fed a normal diet, at that point, they were separated into 6 groups (n = 6). The control group mice were provided with ordinary water and standard chow (Laboratory Rodent Diet 5001 obtained from LabDiet, St. Louis, MO, USA). The high-fat diet comprised of 3% (w/w) cholesterol (Sigma) blended with laboratory high-fat chow (Diet Induced Stoutness Rodent Purified Diet with 60% Energy from Fat). Water was provided ad libitum and after six weeks of high fat feeding exercise was provided from the seventh week. From the seventh week to eighth week, the EX, HF+EX and HF+EX+IF group mice were subjected to swimming for 5 minutes every day, from the ninth weeks to twelfth weeks the animals were subjected to 10 minutes and in the 14^th^ week the animals were subjected to swimming for 15 minutes every day. Animal in HF+IF and HF + EX + IF groups were treated orally with IF (1 mg/kg body Weight) throughout study every day starting post 3 weeks of exercise training. The peptides were diluted in saline by equalizing the protein content in defined volume of aliquots with BSA. The animals were euthanized by decapitation under anesthesia by isoflurane vapor (5%).

### Blood biochemical analysis

The Biochemical analysis was performed with commercially available kits. The GOT (glutamic oxaloacetic transaminase), GPT (glutamic-pyruvic transaminase), investigation was performed using the blood plasma. TG (Triglyceride), TC (Total Cholesterol), LDL-C (low-density lipoprotein cholesterol) and HDL-C (high-density lipoprotein cholesterol) were performed in the serum.

### Tissue staining

The hearts and liver tissues were fixed in formalin, dried through increasing concentrations of alcohol, and embedded in paraffin wax. Two micrometer-thick paraffin slice were cut from paraffin-tissue pieces. The tissue areas were deparaffinized by immersing in xylene also, were rehydrated by decreasing concentration of alcohol. The sections were then stained either for Masson’s trichrome or with hematoxylin and eosin (H and E) and then rinsed with water. Each slide was dried out thoroughly with alcohol. Photomicrographs were obtained using Zeiss Axiophot microscope. For terminal deoxynucleotidyl transferase dUTP nick end labeling (TUNEL), the tissue sections were treated with proteinase K (2 μg/mL) for 15 min and were then washed in PBS twice. The section were then flooded with 0.1% sodium citrate (with 0.1% Triton X-100), absorbed blocking cradle and washed twice with PBS. The sections were subsequently kept in TUNEL reagent (Roche Applied Science, Indianapolis, IN, USA) for 60 min at room temperature. 4,6-diamidino-2-phenylindole (DAPI) reagent was used to stain the nucleus. The nucleus was fluoresced by blue light at 454 nm and the TUNEL-positive cells fluoresced in brilliant green at 460 nm. Photomicrographs were obtained using Zeiss Axiophot microscope.

### Tissue protein extraction and western blotting analysis

Protein extracts from the heart and liver tissue were obtained by homogenization in lysis buffer (100 mg/mL) as mentioned previously [1, 26]. The protein concentrations were determined by Lowry protein assay and the samples were electrophoresed in a 12% SDS polyacrylamide gel electrophoresis (SDS-PAGE), the proteins were then transferred to PVDF membranes (GE Healthcare Life Sciences, Pittsburgh, PA, USA). The membranes were blocked using 5% non-fat milk for 1 h. Monoclonal primary antibodies were diluted 1:1000 in antibody binding buffer (TBS) and used for hybridization overnight (4 °C) with the following antibodies ANP, phosphor-Akt, β-Actin, Bax, BNP, Cytochrome *C*, phosphor-GATA4, PGC1α (sc-20158, sc-7985, sc-47778, sc-526, sc-18818, sc-13560, sc-32823-R, sc-13067, Santa Cruz); phosphor-AMPK, Cle-Caspase-3, phosphor-FOXO3a, Sirt1 (#2535, #9664, #9466, #9475s, Cell Signaling, The Netherlands). Following hybridization with appropriate secondary antibodies the membranes were washed in for 10 min thrice. The blots were detected in chemiluminescent detection using ECL with Fujifilm LAS-3000 (GE Healthcare Life Sciences).

### Statistical analysis

All experimental data were performed from three individual experiments and presented as mean ± S.E.M. One-way ANOVA was performed to compare the statistical difference of all groups. The level of *p* < 0.05 was considered statistically significant.
